# Post-traumatic osteoarthritis following ACL injury

**DOI:** 10.1186/s13075-020-02156-5

**Published:** 2020-03-24

**Authors:** Li-Juan Wang, Ni Zeng, Zhi-Peng Yan, Jie-Ting Li, Guo-Xin Ni

**Affiliations:** 1 School of Sport Medicine and Rehabilitation, Beijing Sport University, Beijing, China; 2 Department of Rehabilitation Medicine, The First Affiliated Hospital of Fujian Medical University, Fuzhou, China

**Keywords:** Post-traumatic osteoarthritis, Anterior cruciate ligament injury, Mechanism, Intervention

## Abstract

Post-traumatic osteoarthritis (PTOA) develops after joint injury. Specifically, patients with anterior cruciate ligament (ACL) injury have a high risk of developing PTOA. In this review, we outline the incidence of ACL injury that progresses to PTOA, analyze the role of ACL reconstruction in preventing PTOA, suggest possible mechanisms thought to be responsible for PTOA, evaluate current diagnostic methods for detecting early OA, and discuss potential interventions to combat PTOA. We also identify important directions for future research. Although much work has been done, the incidence of PTOA among patients with a history of ACL injury remains high due to the complexity of ACL injury progression to PTOA, the lack of sensitive and easily accessible diagnostic methods to detect OA development, and the limitations of current treatments. A number of factors are thought to be involved in the underlying mechanism, including structural factors, biological factors, mechanical factors, and neuromuscular factor. Since there is a clear “start point” for PTOA, early detection and intervention is of great importance. Currently, imaging modalities and specific biomarkers allow early detection of PTOA. However, none of them is both sensitive and easily accessible. After ACL injury, many patients undergo surgical reconstruction of ACL to restore joint stability and prevent excessive loading. However, convincing evidence is still lacking for the superiority of ACL-R to conservative management in term of the incidence of PTOA. As for non-surgical treatment such as anti-cytokine and chemokine interventions, most of them are investigated in animal studies and have not been applied to humans. A complete understanding of mechanisms to stratify the patients into different subgroups on the basis of risk factors is critical. And the improvement of standardized and quantitative assessment techniques is necessary to guide intervention. Moreover, treatments targeted toward different pathogenic pathways may be crucial to the management of PTOA in the future.

## Introduction

Osteoarthritis (OA) is the most common type of arthritis and a leading cause of mobility-related disability, affecting nearly half of the population [1]. It is suspected to be a collection of distinct subtypes, each with a different etiology and clinical characteristics. Classifying OA into multiple disease entities may help to understand its heterogeneity and develop potential interventions targeted toward individual disease processes. Post-traumatic osteoarthritis (PTOA), a subtype of OA, develops after joint injury such as an intra-articular fracture, a ligament injury, or other cartilage (articular or meniscus) injuries within a joint. It accounts for nearly 12% of all cases of symptomatic OA [2]. Unlike idiopathic OA, PTOA represents a cause of functional disability in a disproportionately young population because primary injuries are more likely to be sustained by younger individuals [3, 4]. Besides, PTOA commonly has a known “starting point,” which means that interventions could theoretically be initiated at an early stage to prevent the progression of the disease [3].

Five major risk factors may contribute to PTOA: anterior cruciate ligament (ACL) injury, meniscus tear, glenohumeral instability, patellar dislocation, and ankle instability [1]. Obviously, there are differences between these factors regarding the mechanisms by which the primary joint disorder initiates the subsequent development of OA and the way in which the disease process is maintained. The incidence of ACL injury in particular is high especially in adolescents playing sports that involve pivoting. The reported incidence of PTOA following ACL injury is as high as 87% [5]. This narrative review will outline the incidence of ACL injury that progresses to PTOA, analyze the role of ACL reconstruction in preventing PTOA, suggest possible mechanisms thought to be responsible for PTOA, evaluate current diagnostic methods for detecting early OA, and discuss potential interventions to combat PTOA. Finally, we will identify important directions for future research.

## ACL injury and PTOA

The ACL plays an important role in the stabilization of the knee by restricting anterior translation of tibial and rotational forces at the tibiofemoral joint [6]. As a common orthopedic injury, the annual incidence of isolated ACL injury in the general population is 68.6 per 100,000 people [7]. ACL injury may cause pain, range of motion limitation, muscle weakness, knee instability, altered biomechanics, and reduction in physical activity levels, which place a great economic burden on the health care system [6]. It commonly occurs during sudden deceleration and direction change in non-contact situations [3]. Adolescents and young adults who participate in sports requiring pivoting and frequent direction changes have a high incidence of ACL injury. The risk in young women performing pivoting sports is 3–5 times higher than in men [5].

As reported, 50–90% of ACL injuries progress to PTOA [6]. After ACL injury, grade III or IV radiologic changes in the Kellgren–Lawrence classification system are nearly 5 times more likely than in contralateral knees without a history of ACL injury [8]. A number of factors may mediate the risk of PTOA after ACL injury, such as gender (female), age, high body mass index (BMI), obesity, physical activity level, smoking, low education level, subsequent surgery, time interval between injury and surgery, and varus alignment of the uninjured knee [2, 5, 9, 10].

Older age leads to a disturbance of the balance between anabolic and catabolic processes [5]. Evidence suggests that it is related to medial compartment joint space narrowing [9]. Similarly, BMI is associated with joint space narrowing after ACL injury [5]. Obesity is also believed to have a great influence on OA progress in many ways. One is increased joint loading. Another could be the catabolic effect of inflammatory substances released by adipose tissue, including free fatty acids, reactive oxygen species cytokines, and adipokines on joint tissues. Additionally, obesity is related to increased levels of IL-6 and TNF-α, which are pro-inflammatory indicators of PTOA development [11]. Although the level of physical activity is also considered a risk factor, no consensus has been reached to date. On one hand, physical activity is often recommended to improve function and promote overall health. A lack of mechanical loading contributes to thinning of articular cartilage. A low level of physical activity is associated with a higher BMI, which may lead to the progression of OA. On the other hand, the repetitive use of joints and joint overload may result in matrix loss and chondrocyte apoptosis [2].

## ACL reconstruction and PTOA

Patients who wish to return to high-level activities commonly choose to undergo ACL reconstruction (ACL-R). It is believed that ACL-R helps to restrain the anterior translation of tibia, regain proper joint kinematics, restore knee stability, and prevent excessive torsional loading, thus resulting in pain relief, functional recovery, low complication rates, and highly predictable improvements [1, 11]. Notably, reconstruction methods, including graft choice, attachment point, fixation, and tension, as well as rotational stability, could affect the biomechanical load of the knee joint [1]. Evidence shows that hamstring autografts demonstrate lower incidence, less knee pain, and better self-reported function than bone-patellar tendon-bone autografts [6].

Interestingly, arthroscopic surgery seems to have almost the same incidence as open surgery [6]. However, convincing evidence for the superiority of ACL-R to conservative management in terms of PTOA incidence is still lacking [12, 13]. A number of reasons may explain why ACL-R does not provide protective benefits for long-term joint health. Firstly, surgery cannot completely restore normal joint mechanics [1]. The disruption of normal loading distribution and biomechanics may result in loading on articular areas that are not accustomed to load during weight-bearing activities [14]. Gait analysis reveals that patients with ACL-R knees exhibit altered joint loading patterns and tibial rotation compared with uninjured contralateral knees or healthy patients [14]. The average knee center of rotation (KCOR) during the stance phase of gait after ACL-R changes. Compared with an uninjured contralateral knee, the KCOR of an ACL-R knee is more lateral and anterior at 2 years after surgery, leading to greater motion between the femur and the tibia in the medial compartment relative to the lateral compartment [15]. Secondly, inflammation of the synovium at early time points has been observed [15]. It is supposed that surgery itself could lead to knee joint trauma, and postsurgical hemarthrosis could result in prolonged joint inflammation [13]. Postoperative inflammation may damage synovial stem cells and lead to a compromised joint environment, thus affecting the ability of tissues to heal. A study using mini pigs as animal models showed that the expression of inflammatory cytokines, especially IL-1β, IL-6, and TNF-α, which are correlated with the morphological score of PTOA, increased after an idealized ACL-R [16]. Thirdly, molecular and cellular alterations to joint tissues caused by injury are not readily reversible through joint stabilization [11].

## Early detection of PTOA

PTOA is a progressive pathogenetic process, and it could be too late to intervene when it progresses to a late stage. Therefore, there is a compelling need to improve diagnostic techniques in order to detect PTOA at an early stage. Currently, imaging modalities such as bone scans, radiographs, computed tomography (CT), and magnetic resonance imaging (MRI) and specific biomarkers (biospecimen: blood, serum, synovial fluid, and other tissue samples) [3, 17–19] allow early detection.

Radiography is a commonly used technique to diagnose OA. However, current clinical criteria such as the Kellgren–Lawrence and Outerbridge classification schemes are not sensitive enough to detect early changes of OA, and there is interobserver disagreement when classifying patients [17]. Additionally, radiography is a two-dimensional imaging modality and has limited ability to provide information on ligaments, the synovium, the meniscus, and the articular cartilage [17, 20].

As a repeatable, non-invasive, and multi-planar imaging modality, MRI has been widely used to longitudinally evaluate joint tissues following traumatic injuries [20]. It can perform more sophisticated analysis of various structures within the joint and even quantify the severity of the injury, for instance, characterizing metabolic-triggered subchondral bone damage, evaluating bone marrow lesions, detecting biochemical changes in the cartilage matrix and early cartilage matrix loss, and analyzing cartilage matrix composition [3, 20–22]. T1rho is a technique used to assess proteoglycan content of the extracellular matrix of articular cartilage. And T2 mapping has been used to detect the structural integrity, organization, and water content of cartilage [20]. Physiological MRI has also been used to detect early changes during OA development. Na^18^F positron emission tomography with computed tomography (PET/CT) co-registered with MRI has been demonstrated to be a sensitive imaging modality in an in vivo canine model to detect molecular and cellular changes in bone metabolism before morphological signs appear [4]. The lower delayed gadolinium-enhanced MRI of cartilage (dGEMRIC) index has been shown to have prognostic value for OA development after ACL injury [23]. However, MRI scans are expensive and not available everywhere [17].

A variety of molecular and biochemical processes play important roles in the pathogenesis of PTOA. The detection of molecules in the acute phase provides indications of the future disease process. Breakdown of ECM structures including type II collagen, proteoglycans (PGs), and glycosaminoglycans (GAGs) may be one of the earliest signs of OA and could be detected before radiographic evidence. Elevated concentrations of degradative enzymes for instance matrix metalloprotease (MMP)-1 and MMP-3 in synovial fluid are also measurable after ACL injury. Increased ratio between MMPs and tissue inhibitor of metalloproteinase indicates an increase in degradation relative to synthesis [24]. Given that the alteration of synoviocytes and adjacent chondrocytes may decrease the level of lubricin, the latter is promising as a biomarker of cartilage degradation [3].

## Prevention of PTOA

Obviously, prevention of the initial injury is the most effective tool to manage PTOA since there is still a lack of treatment methods [3, 25]. ACL injury prevention programs play a significant protective role, reducing the incidence of ACL injury by 53% [26]. A systematic review shows that neuromuscular and educational interventions reduce the incidence of ACL injuries by approximately 50% [27]. It has also been suggested that multicomponent injury-prevention training programs, such as strengthening, plyometrics, agility, balance, and flexibility, along with feedback and proper technique for improving lower extremity biomechanics and decreasing landing forces offer great benefits for the protection against ACL ruptures [26]. Prevention programs have been developed for specific sports. For instance, FIFA 11+ is a dynamic field warm-up program designed to decrease the injury risk in soccer. A study demonstrated that its use decreases the rate of ACL injuries in competitive collegiate male soccer players by 77% [28].

## Suggested mechanisms of PTOA after ACL injury

**Fig. 1:**
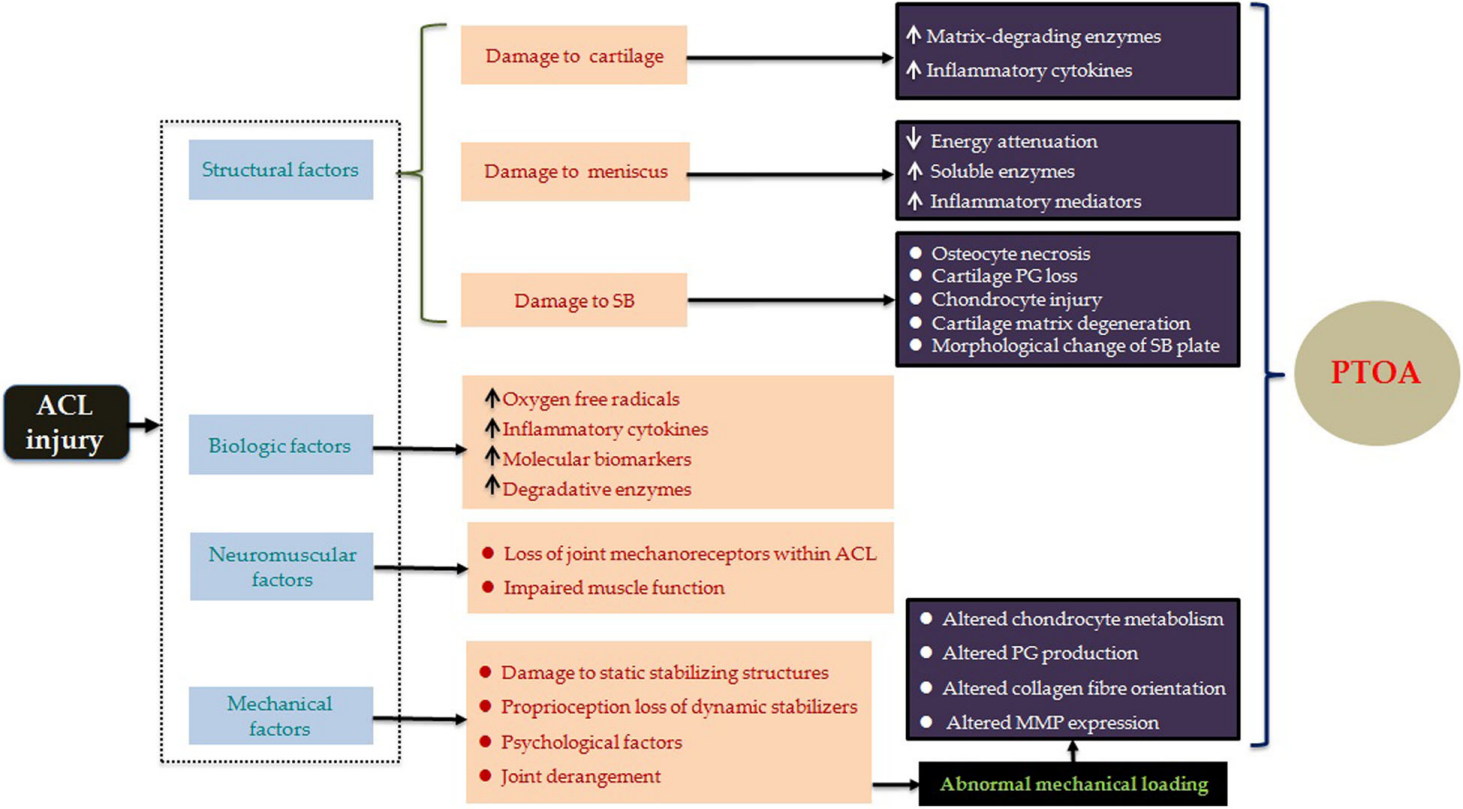
Suggested mechanisms of PTOA after ACL injury. An up arrow (↑) indicates an increase and a down arrow (↓) indicates a decrease

Although accumulating evidence demonstrates that patients with ACL injury are predisposed to PTOA, the precise mechanism remains unclear [6]. Structural, biological, mechanical, and neuromuscular factors are thought to be involved in this process. The involvement of ACL injury in the development of OA may be associated with the mechanisms described in this section (Fig. 1).

### Structural factors

In addition to ACL, many other associated structures may be compromised during initial injury and secondary instability. Compared to patients with isolated ACL rupture, those with concomitant intra-articular injuries have a higher incidence of PTOA [5]. Injury to the articular cartilage (chondral injury), meniscus, ligamentous capsular structures, and subchondral bone [1–3, 5, 6] may contribute to the development of clinically significant OA.

Almost half of patients with ACL injury also suffer from articular cartilage damage of the medial and lateral femoral condyles [3]. Higher impact energy during the initial trauma causes more severe damage to the articular cartilage, with over 25 MPa initiating chondrocyte necrosis and apoptosis [10]. Increased chondrocyte expression of matrix-degrading enzymes and inflammatory cytokines caused by mechanical impact results in chondrocyte apoptosis [1, 3]. As cartilage has a poor healing capacity, damage to the articular chondral surface may directly lead to OA development [5].

One fourth to two thirds of ACL-injured knees have concomitant meniscal damage [16]. It seems that meniscus status is a critically important factor that may contribute to the progression of PTOA. Patients with meniscus tear are more likely to develop radiographic OA compared with patients with isolated ACL injury [29]. Damage to the meniscus decreases the capacity of the joint to attenuate energy. Besides, as a biologically active tissue, the meniscus may synthesize various soluble enzymes and inflammatory mediators in response to trophic trauma that may accelerate the degradation of adjacent cartilage [3].

Notably, 80–90% of patients also show signs of subchondral bone (SB) injuries after ACL injury [5]. When bone marrow lesions are associated with the disruption of adjacent cortical bone and articular surface, they may result in osteocyte necrosis in the bone marrow, significant proteoglycan loss, chondrocyte injury, and matrix degeneration in the overlying cartilage. Subchondral damage is co-localized with bone remodeling, and the balance between bone resorption and formation is disturbed following ACL injury [30, 31]. The alteration of SB mineralization may change the morphology of the SB plate, leading to abnormal mechanical loading on the articular cartilage [31]. These changes in the subchondral bone may initiate the progression to PTOA following ACL injury.

### Biological factors

Following the initial ACL trauma, various biological factors, together with the damage to associated structures, may trigger progressive joint degeneration. Low-grade synovial cellular infiltration, cytokine production, and inflammatory activation of joint tissue cells put patients at risk of progressive OA development [11, 32]. Oxygen free radicals from chondrocytes released during impact injury may lead to progressive chondrocyte damage and matrix degradation. In addition, a large number of cytokines are produced immediately after injury with long-lasting effects, which may disturb homeostasis in the joint and lead to joint degeneration via various metabolic pathways, including inflammatory cytokines IL-1, IL-6, IL-8, IL-17, and TNF-α [3, 5, 12, 33] and molecular biomarkers such as stromal cell-derived factor 1 (SDF-1) and cartilage extracellular matrix fragments [3]. For example, IL-1 downregulates the synthesis of cartilage extracellular matrix (ECM). IL-6 and IL-17 work synergistically with IL-1 to accelerate the breakdown of the ECM. TNF-α plays a role in the increased activity in the apoptotic caspase pathway. The increased levels of IL-1β, TNF-α, and IL-6 are associated with a decreased level of lubricin. Lubricin provides anti-adhesive and chondroprotective properties to the articular cartilage, and the decrease in synovial fluid lubricin following ACL injury increases the risk of degradation [33]. Moreover, these inflammatory biomarkers may stimulate angiogenesis, osteophyte formation, and catabolic enzyme expression [14].

The alteration of gene expression in chondrocytes and the activation of various degradative enzymes, such as MMPs, during injury cause progressive cartilage loss [1, 34]. Increased MMP levels contribute to the degradation of the articular ECM encompassing GAGs, proteoglycans, and collagen, triggering further activation of MMPs, which creates a positive feedback cycle [1]. The loss of proteoglycan and collagen in the articular cartilage is a significant alteration from which it is difficult for the tissue to recover [13]. Increased permeability of the ECM and water content in the articular cartilage induced by catabolic pathways results in alteration of the biochemical and biomechanical properties of the articular cartilage [14].

### Mechanical factors

Mechanical pathways play a vital role in the progression of OA. After injury, an ACL may fail to maintain the joint as stably as before. Consequently, chronic changes in the static and dynamic loading of the knee may lead to the degradation of the cartilage and other joint structures [5]. Reasons that could contribute to abnormal mechanical loading of knee joints include damage to static stabilizing structures, proprioception loss of dynamic stabilizers such as quadriceps and hamstrings, psychological factors such as emotional distress caused by pain and fear of re-injury, residual muscle weakness and disuse atrophy [1], joint derangement, and biomechanical variables [35]. Adaptive changes during ambulation due to mechanical factors may lead to the disruption of joint homeostasis [36]. Given that chondrocytes are very sensitive to mechanic environment alterations, abnormal mechanical loading caused by various factors could change chondrocyte metabolism, proteoglycan production, collagen fiber orientation, and MMP expression, lead to ECM degradation, alter the mechanical properties of the cartilage itself, and ultimately cause functional disability [1, 3].

Kinematic abnormalities and kinetic alteration following joint injury are associated with OA development [37]. Knee joint structures, such as the ACL, the medial collateral ligament, and the lateral collateral ligament, work synergistically to limit the motion of anterior tibial translation. In patients with ACL injury, load is distributed to other structures to compensate for ACL deficiency [15].

### Neuromuscular factors

Impairment of neuromuscular functions may also contribute to the development of PTOA [5, 6]. The alteration of neuromuscular feedback caused by persistent ligament laxity [14] and impaired muscle function [10] poses a risk of progressive degradation of structures within the joints. The ACL not only restricts tibiofemoral motion passively but also serves as a dynamic sensor transmitting afferent information to the central nervous system. The loss of joint mechanoreceptors within the ACL after traumatic injury results in altered information input, decreased motor output, and poor neuromuscular control [38]. Patients with ACL injury suffer from quadriceps and hamstring strength deficit due to disuse atrophy or arthrogenic muscle inhibition. As shock absorbers and dynamic stabilizers, the quadriceps distribute load across the articular surface and stabilize the knee joint [38, 39]. When the quadriceps are weak, articular loading of the knee joint increases, which may initiate a degenerative process [2, 40].

## Treatment of PTOA

As a progressive and chronic condition, PTOA should be treated at an early stage to minimize its long-term effects and prevent the development of end-stage OA [3, 15]. Unlike idiopathic OA, there is a clear injurious event involved in the case of PTOA. A known “starting point” presents the opportunity for targeted treatments [3]. Intervening immediately after injury plays an important role in the prevention of future degradation.

A better understanding of pathogenic pathways makes it possible to develop targeted interventions to prevent clinically significant disease. The most discussed treatment methods in the literature are biological interventions, including anti-cytokine and chemokine interventions (intraarticular injection of IL-1Ra) [5, 11], anti-resorptive therapies (bisphosphonates and strontium ranelate, etc.) [35], anti-oxidant treatment (methylsulfonylmethane and pycnogenol, etc.) [18], and joint aspiration to remove hemarthrosis at the time of injury [17]. Selective inhibition of IL1, IL6, IL-17, and metalloproteinases may decrease the degradation of the articular cartilage ECM, and inhibition of resistin and TNF-a may decrease synovial inflammation and boundary lubrication [3]. Extracellular matrix–blood composite injection relieved the pain during weight bearing and attenuated cartilage damage after ACL transection in a rat model [37]. Studies on genetically engineered mice show promising interventions targeting certain gene transcriptions [3]. Using p16-3MR transgenic mice, Jeon et al. [41] demonstrated that selective removal of senescent cells retards OA progression, reduces pain, and creates a pro-regenerative environment. Non-pharmacological treatments such as cryotherapy improve footprint patterns and reduce synovial inflammation [42]. Weight loss is often recommended in treatment programs, as it decreases joint loading and IL-7 levels through biomechanical and inflammatory pathways [11].

Surgical techniques such as reconstructive procedures have been improved and new ones, such as arthroscopic surgery, have been introduced. As mentioned earlier, the use of hamstring autografts in ACL-R exhibits good clinical outcomes [6]. Notably, the functional outcome in quadriceps autograft groups is equal to or better than in hamstring autograft groups [43]. For patients with concomitant meniscus injury, the more of the meniscus is preserved, the better the outcome will be. Thus, meniscal repair instead of partial meniscectomies during surgery is recommended [5]. Removing a part of the meniscus decreases the distribution of the transmission force, and partial meniscectomy decreases quadriceps strength, which could be associated with altered lower extremity biomechanics [6].

Regardless of treatment by surgical or nonsurgical means, an integrated rehabilitation program that helps to improve neuromuscular control, strength, power, and muscular symmetry is necessary [1, 5]. Rabbits treated with early continuous passive motion (CPM) after ACL transection have normal articular surfaces, thicker articular cartilage, better tidemark continuity, lower levels of inflammatory cytokine, and abundant GAG, indicating that CPM has a significant effect in protecting against PTOA [44]. In Frobell’s study, for young patients with acute ACL tear, structured rehabilitation plus early ACL-R did not result in better outcome of Knee Injury and Osteoarthritis Outcome Score than patients with structured rehabilitation plus optional delayed ACL-R. Moreover, for patients using latter strategy, 61% of ACL-R could be avoided without adverse outcome, indicating that structured rehabilitation program is of great importance in the management of ACL injury [45]. For patients undergoing ACL-R, early rehabilitation both preoperatively and postoperatively is needed [5]. Exercise is an integral part of rehabilitation programs and has positive effects [46]. Patients with limited range of motion have a higher incidence of PTOA. Those with a quadriceps and hamstring strength deficit fail to maintain normal loading patterns and absorb impact, which may lead to joint space narrowing. Therefore, returning to the normal range of motion and quadriceps and hamstring strength training should be essential parts of rehabilitation programs [14].

Education also plays a crucial part that cannot be ignored. It is necessary to raise patients’ awareness of re-injury and PTOA risk, help them understand the importance of re-injury prevention and return-to-play criteria, and educate them in modifying physical activity and weight management and diet.

## Conclusions and future directions

Patients with ACL injury have a high risk of developing PTOA [5, 6]. Although much work has been done, the incidence of PTOA among patients with a history of ACL injury remains high due to the complexity of ACL injury progression to PTOA, the lack of sensitive and easily accessible diagnostic methods to detect OA development, and the limitations of current treatments [1].

PTOA development is a chronic and progressive condition. At its late stages, the changes in the knee joint are irreversible, and arthroplasty might be the only treatment choice [17, 34]. Therefore, early detection and assessment of OA severity is necessary to guide therapy and prevent irreparable damage to the knee joint [34]. Though detection methods such as imaging modalities and biomarkers now exist, none of them are both sensitive and easily accessible [3, 17]. Future research directions should be the improvement of standardized and quantitative assessment techniques to detect PTOA at an early stage, monitor the progression and severity of OA development, and evaluate the efficacy of treatments.

At present, the way to prevent the progression of PTOA remains unclear, as a number of risk factors may be at play [25]. Animal models and experiments in vivo allow the study of pathological pathways triggered by ACL injury [19, 47]. A complete understanding of its mechanisms to classify patients into different subgroups on the basis of risk factors is critical [3]. Treatments targeted toward different pathogenic pathways may be key to the management of PTOA in the future.

Unlike idiopathic OA, PTOA has a clear “starting point,” which makes it possible to trace pathological pathways and intervene immediately after injury [37]. The improvement of conservative, surgical, and pharmacological treatments to diminish the deleterious impact of inflammation, delay PTOA, and promote optimal long-term health after ACL injury is imperative. An animal study shows that controlling knee stability plays a protective role in OA development [48]. After ACL injury, surgery to restabilize the knee joint is often recommended to mitigate knee rotational instability, restrain tibia anterior translation, and restore function [6, 8, 9]. The improvement of surgical techniques allows for better clinical outcomes. Novel approaches such as bio-enhanced ACL repair have been developed to produce similar structural properties in ACL grafts and provide protection of the articular cartilage in a porcine model [49]. Anatomic ACL-R with a minimum ACL-R scoring checklist score of 8 indicates a reduced incidence of OA compared with non-anatomic ACL-R in a minimum 10-year follow-up [50]. Thus, advanced anatomical reconstruction techniques should be developed to restore normal mechanics and reduce the risk of OA [5].

The effect of pharmacological treatment has also been widely investigated in animal studies. As our knowledge of biological mechanisms triggered by ACL injury increases, selective inhibition of inflammatory chemokines such as IL-1 and TNF-α has shown potential for preventing the degradation of injured joints in animal studies [3]. Intra-articular injection of dexamethasone has been shown to decrease joint swelling, suppress catabolic gene expression, lower the histological grade, and reduce the formation of osteophytes in rabbit models [13, 32]. AMD3100 can prevent trabecular bone loss and mitigate cartilage degeneration in PTOA mice by inhibiting the SDF-1α/CXCR4 signaling pathway [27]. Intra-articular injection of triamcinolone acetonide after ACL transection attenuates synovitis and collagen degradation in Yorkshire pigs [51]. Further work is warranted for clinical application of targeted therapy.

In future studies, standardized criteria should be developed to determine whether a patient needs to receive conservative treatment or surgery reconstruction, which may reduce the financial burden on the health care system and prolong joint health [6]. Finally, a better understanding of the course of specific inflammatory chemokine production and healing processes is of crucial importance in determining the duration of intervention.

## Data Availability

Not applicable.

## Abbreviations

OA: Osteoarthritis
PTOA: Post-traumatic osteoarthritis
ACL: Anterior cruciate ligament
ACL-R: ACL reconstruction
MRI: Magnetic resonance imaging
ACL-D: ACL-deficit
KCOR: Knee center of rotation
BMLs: Bone marrow lesions
SDF-1: Stromal cell-derived factor 1
ECM: Extracellular matrix
MMPs: Matrix metalloproteases
CT: Computed tomography
PET/CT: Positron emission tomography with computed tomography
dGEMRIC: Delayed gadolinium-enhanced MRI of cartilage
GAG: Glycosaminoglycan
